# Radiomics Signature Facilitates Organ-Saving Strategy in Patients With Esophageal Squamous Cell Cancer Receiving Neoadjuvant Chemoradiotherapy

**DOI:** 10.3389/fonc.2020.615167

**Published:** 2021-02-19

**Authors:** Yue Li, Jun Liu, Hong-xuan Li, Xu-wei Cai, Zhi-gang Li, Xiao-dan Ye, Hao-hua Teng, Xiao-long Fu, Wen Yu

**Affiliations:** ^1^Department of Radiation Oncology, Shanghai Chest Hospital, Shanghai Jiao Tong University, Shanghai, China; ^2^Shanghai Jiao Tong University School of Medicine, Shanghai, China; ^3^Department of Thoracic Surgery, Shanghai Chest Hospital, Shanghai Jiao Tong University, Shanghai, China; ^4^Department of Radiology, Shanghai Chest Hospital, Shanghai Jiao Tong University, Shanghai, China; ^5^Department of Pathology, Shanghai Chest Hospital, Shanghai Jiao Tong University, Shanghai, China

**Keywords:** neoadjuvant chemoradiation, esophageal cancer, response prediction, organ-saving treatment, radiomics

## Abstract

After neoadjuvant chemoradiotherapy (NCRT) in locally advanced esophageal squamous cell cancer (ESCC), roughly 40% of the patients may achieve pathologic complete response (pCR). Those patients may benefit from organ-saving strategy if the probability of pCR could be correctly identified before esophagectomy. A reliable approach to predict pathological response allows future studies to investigate individualized treatment plans.

**Method:**

All eligible patients treated in our center from June 2012 to June 2019 were retrospectively collected. Radiomics features extracted from pre-/post-NCRT CT images were selected by univariate logistic and LASSO regression. A radiomics signature (RS) developed with selected features was combined with clinical variables to construct RS+clinical model with multivariate logistic regression, which was internally validated by bootstrapping. Performance and clinical usefulness of RS+clinical model were assessed by receiver operating characteristic (ROC) curves and decision curve analysis, respectively.

**Results:**

Among the 121 eligible patients, 51 achieved pCR (42.1%) after NCRT. Eighteen radiomics features were selected and incorporated into RS. The RS+clinical model has improved prediction performance for pCR compared with the clinical model (corrected area under the ROC curve, 0.84 vs. 0.70). At the 60% probability threshold cutoff (i.e., the patient would opt for observation if his probability of pCR was >60%), net 13% surgeries could be avoided by RS+clinical model, equivalent to implementing organ-saving strategy in 31.37% of the 51 true-pCR cases.

**Conclusion:**

The model built with CT radiomics features and clinical variables shows the potential of predicting pCR after NCRT; it provides significant clinical benefit in identifying qualified patients to receive individualized organ-saving treatment plans.

## Introduction

Neoadjuvant chemoradiotherapy (NCRT) followed by esophagectomy has significantly improved the survival of resectable locally advanced esophageal cancer compared with surgery alone and has been established as the standard treatment (1, 2). Although the response to NCRT varies among patients, the pathologic complete response (pCR) rate can be as high as 43.2% in esophageal squamous cell carcinoma (ESCC) and 27% in esophageal adenocarcinoma (1–4). For patients who achieve pCR after NCRT, individualized organ-saving strategies such as active surveillance or definitive chemoradiation are recently being explored as an alternative treatment option to surgery, considering the relatively high postoperative complication rate (~65%) and mortality rate (~4–10%) depending on different centers (5, 6), as well as the decline in health related quality of life after esophagectomy (7–10). However, pCR could only be confirmed by histologic assessment of surgical specimens. A reliable means independent from surgical specimen evaluation is required to identify the complete responders that could potentially spare surgery. Current recommended approaches for NCRT response assessment include pathologic evaluation of endoscopic biopsy and ^18^FDG-PET that usually involves setting a cutoff value of SUV reduction to discriminate between pCR and non-pCR patients. However, those approaches are not accurate enough to identify pCR patients; thus, some non-pCR patients might be falsely diagnosed as complete responders and inappropriately arranged for surgery omission (11). So far, no biological or radiological marker has been used for guiding the comprehensive esophagus-preserving treatment modality in locally advanced esophageal cancer.

Radiomics is the high-throughput extraction of a large amount of image features (density, grey level heterogeneity, shape, etc.) from radiographic images that are promising in revealing the underlying proteo-genomic and phenotypic information of solid tumors (12). While the histopathologic analysis of biopsy specimens might fail to represent the whole tumor due to the spatial heterogeneities, radiomics is able to profile these heterogeneities and serves as a bridge between tumor genomics and phenotypes. Some radiomics features have been proved to correspond to the gene expression profile and are useful in predicting cancer prognosis and therapeutic response (13). Radiomics features extracted from ^18^FDG-PET images combined with clinical information was reported to have decent discriminatory accuracy in predicting pCR in post-NCRT esophageal tumors with AUC (area under the receiver operating characteristic curve) of 0.81 (14). However, the investigation was performed mainly for tumors of gastroesophageal junction or esophageal adenocarcinoma, and the conclusion could not be extended to ESCC, the type that predominates in Asian countries. Therefore, we aim to develop a CT radiomics based model to predict tumor response to NCRT in ESCC and assess its value in organ-saving decision making.

## Materials and Methods

### Patients

This retrospective study was approved by the institutional review board of Shanghai Chest Hospital; the requirement for informed consent was waived. Consecutive patients with stage T2-4aN+/-M0 esophageal cancer who received NCRT followed by esophagectomy in Shanghai Chest Hospital from June 2012 to June 2019 were extracted from the hospital database. Patients are only eligible for inclusion if they (i) had histopathologically confirmed ESCC; (ii) had contrast-enhanced CT scans within 3 weeks before NCRT and within 3–8 weeks after NCRT. Patients were excluded if (i) the chemoradiation was done outside Shanghai Chest Hospital, and the treatment details were missing; (ii) delivered radiation dose was less than 40 Gy or more than 50.4Gy; (iii) surgery was done within less than 4 weeks or more than 10 weeks after NCRT—indicating urgent and salvage resections, respectively (2, 3).

### Histopathological Assessment

Surgically resected specimens were sent for histopathological assessment by an experienced pathologist and reviewed by another specialized thoracic cancer pathologist. Pathologic complete response (pCR) was defined as the absence of microscopically viable cancer cells in the primary tumor, as opposed to any grade of residual carcinoma (Non-pCR). Evaluation of lymph node metastasis was excluded because radiomics analysis is unreliable when performed on small lesions, and thus only the primary tumor would be involved in the image analysis (3).

### Clinical Variable Collection

Demographic information and radiologic test results from CT, EUS (endoscopic ultrasound), and esophagogram were collected as clinical variables. Clinical T stage and lymph node status (N+/N-) were evaluated by EUS and CT complementarily. δThickness% was calculated as the maximum tumor thickness reduction after NCRT divided by baseline maximum tumor thickness on pre-NCRT CT. Tumor adventitia type was evaluated by CT and classified as smooth or not smooth (tumor outer membrane is coarse or nodular) (15). Esophagogram esophageal cancer gross type was classified as 4 types according to Japan Esophageal Society described as following: type 1: protruding type; type 2: ulcerative and localized type; type 3: ulcerative and infiltrative type; type 4: diffusely infiltrative type (16, 17). Pre-Dmin and post-Dmin refer to the esophageal minimum diameter on esophagogram before and after NCRT, respectively. δDmin% was defined as the increase of esophageal minimum diameter on esophagogram after NCRT divided by pre-Dmin. The difference of clinical variables between pCR and non-pCR cohorts was analyzed using Chi-squared test or Student t-test, and only the significant clinical variables were selected for further analysis.

### Delineation of Regions of Interest

Contrast-enhanced chest CT images were acquired with a variety of CT scanners according to standard clinical scanning protocols (120kV/140kV, 140~300mA, and slice thickness of 5 mm). All images were reconstructed with the standard reconstruction kernel. The regions of interest (ROIs) were manually delineated on Pinnacle 9.1 system (Philips, Fitchburg, WI) by two expert radiation oncologists, referring to complementary materials such as ^18^FDG-PET/CT, barium esophagogram, and esophagoscopy reports. The pre-NCRT ROI was contoured on the pre-NCRT CT images to cover the primary esophageal tumor only. The post-NCRT CT images of each patient were then registered with the corresponding pre-NCRT images, and the contour of the pre-NCRT ROI was projected onto the post-NCRT images. The post-NCRT ROI was manually adjusted from the pre-NCRT ROI to compensate for the circumferential tumor shrinkage after treatment, keeping the craniocaudal length unchanged.

### Radiomics Feature Extraction

Radiomics features were extracted using the open infrastructure quantitative image software IBEX (18). A total of 135 radiomics features were extracted from both pre-NCRT and post-NCRT CT images, respectively, including 18 shape and size based features, 52 first order statistic features, and 65 second order features (**Supplementary Material 1**).

For each of these radiomics features, δ-NCRT feature was calculated as the post-NCRT radiomics feature value subtracting the corresponding pre-NCRT one, producing 135 δ-NCRT features. Therefore, a total of 405 features would be extracted for each patient.

### Feature Reproducibility Evaluation

To assess the inter-observer reproducibility of radiomics features, the pre-NCRT CT images of the first 10 consecutive patients were used, each contoured by another two experienced thoracic cancer radiation oncologist in a blinded fashion. The intraclass correlation coefficient (ICC) was calculated for the feature robustness ranking. The coefficients were interpreted as follows: 0.81 to 1.00: almost perfect agreement; 0.61 to 0.80: substantial agreement; 0.41 to 0.60: moderate agreement; 0.21 to 0.40: fair agreement; 0 to 0.20: poor or no agreement. The feature stability was also validated in test-retest setting using RIDER dataset from The Cancer Imaging Archive (TCIA), which contains two sets of CT scans taken 15 min apart for each of the 31 NSCLC patients. The repeatability in test-retest was evaluated by concordance correlation coefficient (CCC). The radiomics features with both ICCs above 0.4 in inter-observer test and CCCs above 0.75 in test-retest were selected for further analysis (19, 20).

### Radiomics Feature Selection

Radiomics feature selection was performed in two steps. Robust features selected from reproducibility analysis were first tested by univariate logistic regression with a cutoff p-value of 0.157 according to Wilks’ theorem and Akaike Information Criterion requiring χ ^2^ >2 df, where df is degrees of freedom (14). The significant features were then introduced into a regularized multivariate logistic regression with the least absolute shrinkage and selection operator (LASSO) penalty, which shrinks the estimates of regression coefficients and excludes variables by forcing certain coefficients to become 0. The purpose of this shrinkage is to prevent overfitting due to either collinearity of the covariates or high-dimensionality (21). A radiomics signature (RS) was constructed through linear combination of the selected radiomics features weighted by their coefficients in LASSO regression. Student t-test was performed to evaluate the mean difference of RS between pCR and non-pCR cohorts.

### Model Development and Statistical Analysis

Two multivariate logistic regression models were constructed to study the value of clinical variables alone (clinical model) and the added value of radiomics signature (RS+clinical model), for the prediction of pCR. The flowchart of the model development process is attached in the **Supplementary Materials**.

The goodness-of-fit of each model was assessed by Nagelkerke R^2^, Akaike Information Criterion (AIC), and Brier score. The lower the AIC value and Brier score are, the better the model fits: for a binary outcome, the Brier score ranges from 0 for a perfect model to 0.25 for an unsatisfying model (22). On the contrary, higher Nagelkerke R^2^ indicates better calibration. Model calibration was visualized by the calibration plot. Discriminative ability of the models was evaluated by area under the receiver operating characteristic (ROC) curve (AUC).

Considering the traditional accuracy metrics, such as AUC, have limited value for telling if an intervention could be performed on the individual patient, decision curve analysis was carried out to investigate the clinical usefulness of the prediction models by quantifying the net benefit, which is calculated as (23, 24):

$$net\;benefIt=\frac{TP}{n}-\frac{FP}{n}\left(\frac{P_{t}}{1-P_{t}}\right)$$

where TP and FP refer to true positive count (i.e., true pCR) and false positive count (i.e., false pCR); n is the number of total patients; and P_t_ is the threshold probability. Threshold probability is defined as the minimum probability of pCR above which a patient would opt for observation rather than surgery (higher probability indicates a greater chance of pCR). Finally, a nomogram incorporating the selected clinical variables and RS was generated for clinical reference.

To prevent the overestimation of the final model performance, internal validation by bootstrap resampling with 2,000 replicates was performed to correct the optimism of the model performance.

Statistical analysis was done with R (version 3.6.1) and p-value less than 0.05 was considered significant unless stated otherwise.

## Results

### Patient Characteristics and Clinical Variable Selection

A total of 121 patients with ESCC were finally included in the study with an average age of 60.9 (± 6.8) years and more males (88.4%) than females (11.6%).The clinical characteristics are shown in **Table 1**.

**Table 1:** Clinical characteristics of patients in pCR and non-pCR cohorts.

Characteristic	pCR (n=51)	non-pCR (n=70)	p-value
**Sex**			0.390
** Female**	4(7.8%)	10(14.3%)	
** Male**	47(92.2%)	60(85.7%)	
**Age (years)^#^**	62.6(± 6.9)	59.6(± 6.5)	0.018*
**Smoking history**			0.580
** Yes**	26(51.0%)	31(44.3%)	
** No**	25(49.0%)	39(55.7%)	
**Alcohol history**			0.190
** Yes**	24(47.1%)	24(34.3%)	
** No**	27(52.9%)	46(65.7%)	
**Radiation dose**			0.590
** 40Gy**	9(17.6%)	16(22.8%)	
** 41.4Gy**	39(76.5%)	52(74.3%)	
** 50.4Gy**	3(5.9%)	2(2.9%)	
**Chemotherapy regimen**			0.338
** PF**	17(33.3%)	16(22.9%)	
** TC/TP**	22(43.1%)	39(55.7%)	
** Others**	12(19.6%)	15(21.4%)	
**Tumor location**			0.943
** Upper thoracic**	7(13.7%)	9(12.9%)	
** Middle thoracic**	21(41.2%)	31(44.3%)	
** Lower thoracic**	23(45.1%)	30(42.8%)	
**Clinical T stage**			0.144
** 2**	4(7.8%)	9(12.8%)	
** 3**	36(70.6%)	37(52.9%)	
** 4a**	11(21.6%)	24(34.3%)	
**Clinical N status**			0.351
** N+**	27(52.9%)	43(61.4%)	
** N-**	24(47.1%)	27(38.6%)	
**Tumor length^#^**			
** By EUS (mm)**	63.0(± 28.0)	42.3(± 77.1)	0.085
** By esophagogram (mm)**	62.3(± 21.9)	61.6(± 20.8)	0.855
** By CT (mm)**	64.1(± 22.7)	62.9(± 20.0)	0.750
**Pre-thickness by CT (mm)^#^**	20.7(± 5.3)	21.1(± 5.8)	0.710
**Post-thickness by CT (mm)^#^**	11.7(± 3.4)	14.0(± 4.6)	0.004*
**δThickness by CT (mm)^#^**	9.0(± 5.0)	7.1(± 5.5)	0.056
**δThickness% by CT (%)^#^**	41.4(± 17.3)	31.4(± 19.6)	0.004*
**CT Advantitia type**			0.044*
** Smooth**	37(72.5%)	39(55.7%)	
** Not smooth**	14(31.1%)	31(68.9%)	
**Gross type by esophagogram**			0.760
** Type 1**	12(24.5%)	15(23.4%)	
** Type 2**	8(16.3%)	11(17.2%)	
** Type 3**	22(44.9%)	24(37.5%)	
** Type 4**	7(14.3%)	14(21.9%)	
** Not available**	8	–	
**Pre-Dmin by esophagogram(mm)^#^**	8.8(± 3.3)	8.5(± 3.6)	0.670
**Post-Dmin by esophagogram(mm)^#^**	10.8(± 3.0)	9.4(± 3.1)	0.012*
**δDmin by esophagogram(mm)^#^**	3.8(± 3.2)	3.1(± 2.2)	0.270
**δDmin% by esophagogram (%)^#^**	49.9(± 40.7)	64.3(± 101.7)	0.430

*p<0.05

^#^Expressed as mean±SD.

Data are numbers, with percentages in parentheses.

pCR: pathologic complete remission.

NP(vinorelbine plus cisplatin), DP(docetaxel plus cisplatin), or SP(oral tegafur-gimeracil-oteracil potassium capsule (s-1) administered with intravenous cisplatin) regimen.

Tumor thickness was defined as the maximum thickness on CT;

Tumor gross type by esophagogram was classified according to Japan Esophageal Society standard;

Dmin refers to the esophageal minimum diameter on esophagogram.

All patients received full prescribed radiation dose, including 40Gy in 20 fractions, 41.4Gy in 23 fractions, or 50.4Gy in 28 fractions, which was delivered 5 times a week over a duration of 4–6 weeks. Concurrent chemotherapy regimens administered to patients included PF regimen (5-fluorouracil plus cisplatin), TC/TP (paclitaxel administered with cisplatin or carboplatin), SP (oral tegafur-gimeracil-oteracil potassium capsule [s-1] administered with intravenous cisplatin), NP (vinorelbine plus cisplatin), and DP (docetaxel plus cisplatin). All patients completed full cycles of concurrent chemotherapy except 4 (3.3%) due to myelosuppression or unfavorable nutritional status. After NCRT, 51 patients (42.1%) achieved pCR.

As shown in **Table 1**, older patients and those with a smooth tumor adventitia type on CT was prone to respond better to NCRT. Both post-thickness and δthickness% had significant association with pCR, which was confirmed by p-values of 0.004 from t-test, indicating that a better post-NCRT tumor regression was correlated with a higher chance of pCR. Apparent multicollinearity was found between these two features (Pearson correlation coefficient, 0.92), and δThickness% was selected over post-thickness due to its superior significance in univariate logistic test (p-value, 0.005 vs. 0.059). Furthermore, a larger post-Dmin by esophagogram, indicating a better restoration of esophageal dilatation after NCRT, was significantly associated with pCR. As a result, four significant clinical variables, including age, tumor adventitia type, δthickness%, and post-Dmin by esophagogram, were selected to enter the prediction model.

### Radiomics Feature Selection

**Figure 1:**
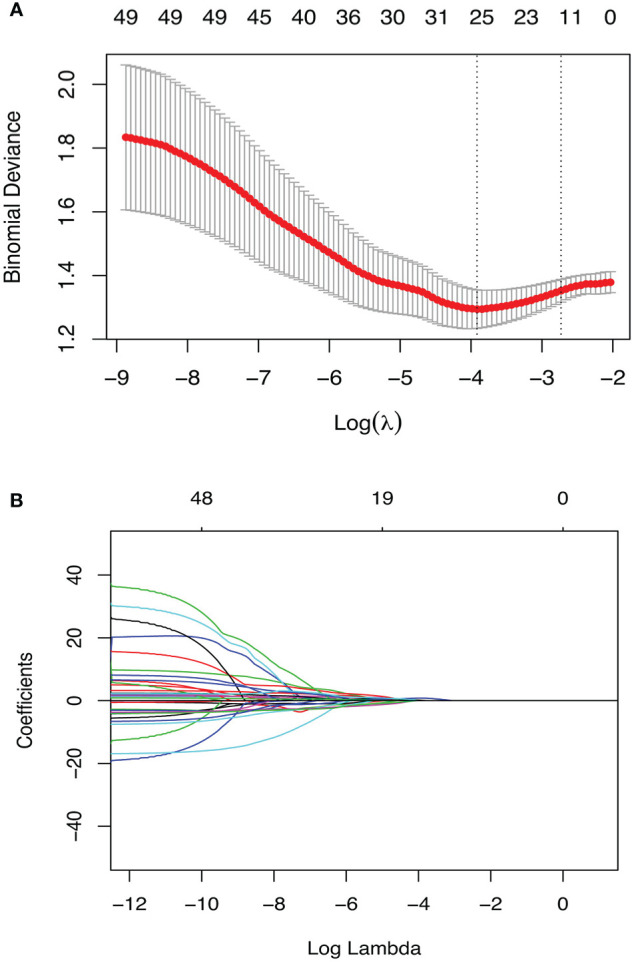
Radiomics feature selection using the penalized logistic regression model with LASSO penalty. **(A)** The tuning parameter lambda(λ) selection with 10 folds cross-validation and binomial deviance curve was plotted against log(λ). The selected model was built with λmin(0.020), equivalent to log(λ) = -3.92. **(B)** Lasso regression coefficients profile. Coefficients are plottted against log(λ) depicting the trend of approaching zero as λ increase.

Of the 135 radiomics features, 93 showed at least moderate inter-observer reproducibility (intraclass correlation coefficient, ICC>0.4); 116 features showed good test-retest repeatiblity (concordance correlation coefficient, CCC>0.75), and a total of 89 features were on the intersection of the above two groups (**Supplementary Material 2**). Hence, 267 radiomics features (89 pre-NCRT, 89 post-NCRT, and 89 δ-NCRT features) were introduced into the following feature selection process. Of these robust radiomics features, 49 were significant in univariate logistic regression analysis (p-value<0.157) (**Supplementary Material 3**), among which 18 features were further selected by the regularized multivariate logistic regression model with LASSO penalty (**Figures 1A, B**), including 5 pre-NCRT, 7 post-NCRT, and 6 δNCRT radiomic features (see **Supplementary Material 4, 5**). None of the selected radiomics feature is correlated with δthickness% or post-Dmin. Radiomics signature (RS) of the pCR cohort was significantly higher than that of the non-pCR cohort by t-test (0.25 0.95 vs. -0.82 0.84, p= 3.77E-09).

### Model Development and Model Performance

**Table 2** shows parameters of the two prediction models for pCR fitted with multivariate logistic regression (probability formulas are presented in **Supplementary Material 7**). Four clinical variables significantly associated with pCR (age, tumor adventitia type, δthickness%, and post-Dmin) were incorporated in the clinical model. RS was added to the clinical model to develop the RS+clinical model.

**Table 2:** Two prediction models for pCR using multivariate logistic regression.

Features	Clinical model		RS+clinical model	
	OR(95% CI)	p-Value	OR(95% CI)	p-Value
**Age**	1.08(1.02,1.16)	0.018	1.12(1.03,1.21)	0.007
**δThickness%**	1.02(1.00,1.05)	0.036	1.01(0.98,1.03)	0.588
**Post-Dmin**	1.12(0.97,1.30)	0.129	1.08(0.91,1.28)	0.408
**Adventitia type**	2.78(1.16,7.12)	0.026	4.74(1.68,15.10)	0.005
**RS**	–	–	5.06(2.72,10.60)	0.000

95% CI: 95% confidence interval.

Age is counted in years.

OR, odds ratio. The estimated pCR odds increase corresponds to the increment of the continuous variables by the following units: 1 year for age, 1% for δThickness%, 1 mm for post-Dmin, and 1 unit for RS.

RS, Radiomics signature.

**Figure 2:**
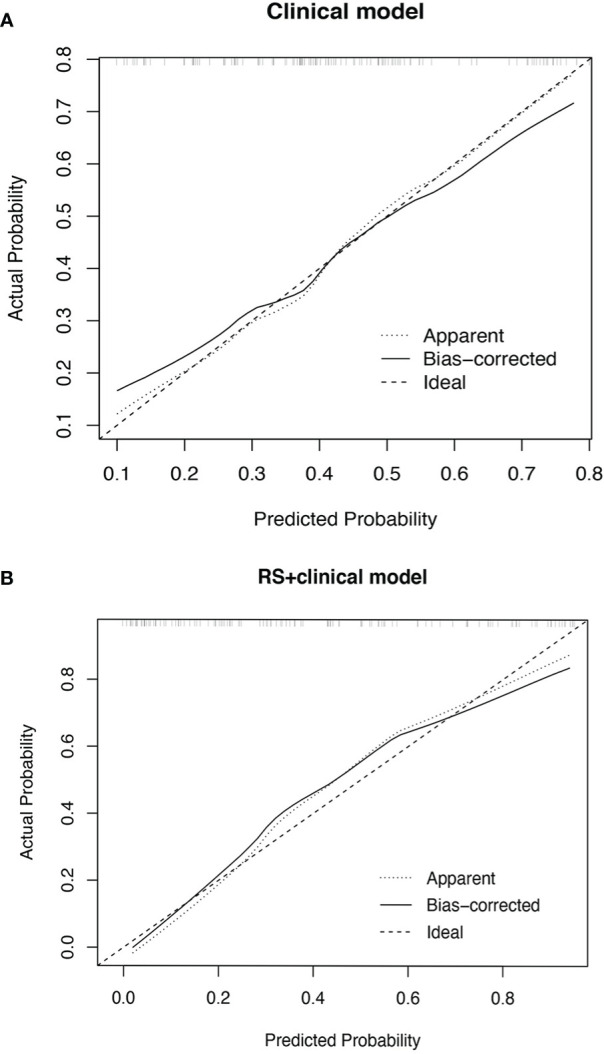
Calibration plot of the clinical model **(A)** and RS+clinical model **(B)**. The calibration curves of clinical model and RS+clinical model showing the difference between the predicted probability of pCR and the observed (actual) probability. The “Ideal” line represents the perfect prediction as the predicted probabilities equal to the observed probabilities. The “Apparent” curve is the calibration of the primary cohort. The “Bias-corrected” curve was the calibration created by internal validation of 2000-replicate bootstrap on the primary cohort.

The performance measures of two models are displayed in **Table 3**. RS+clinical model exhibited a better goodness-of-fit than the clinical model (Nagelkerke R^2^: 0.50 vs. 0.21; AIC: 120.88 vs. 153.79; Brier score: 0.15 vs. 0.20) and was better calibrated than the clinical model (**Figure 2**).

**Table 3:** Performance of prediction models.

Model	Goodness-of-fit			Discrimination	Corrected performance	
	Nagelkerke R^2^	AIC	Brier score	AUC	Internal Validated Nagelkerke R^2^	Internal Validated AUC
**Clinical model**	0.21	153.79	0.20	0.73	0.25	0.70
**RS+clinical model**	0.50	120.88	0.15	0.87	0.43	0.84

AUC, Area under the receiver operating characteristic (ROC) curve.

AIC, Akaike Information Criterion.

Internal validation was performed with 2,000-replicate bootstrapping on the primary cohort.

**Figure 3:**
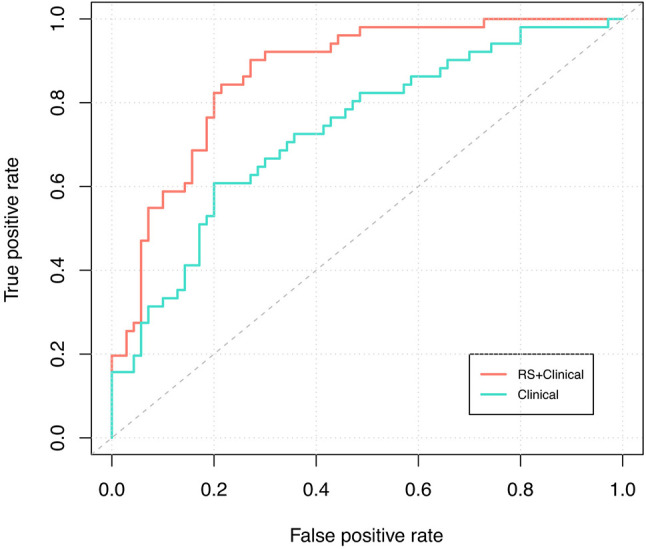
ROC curve analysis. Receiver-operating-characteristic cuve analysis of the two models indicating their ability to discriminate between pCR and non-pCR patients. The blue line represents the ROC curve of the clinical model and the corrected AUC is 0.70; the red line represents the ROC curve of the RS+clinical model and the corrected AUC is 0.84.

RS+clinical model also demonstrated a superior discriminative performance than the clinical model (AUC: 0.87 vs. 0.73), and this advantage persisted after internal validation (corrected AUC, 0.84 and 0.70; **Figure 3**).

### Clinical Benefit and Nomogram

**Figure 4:**
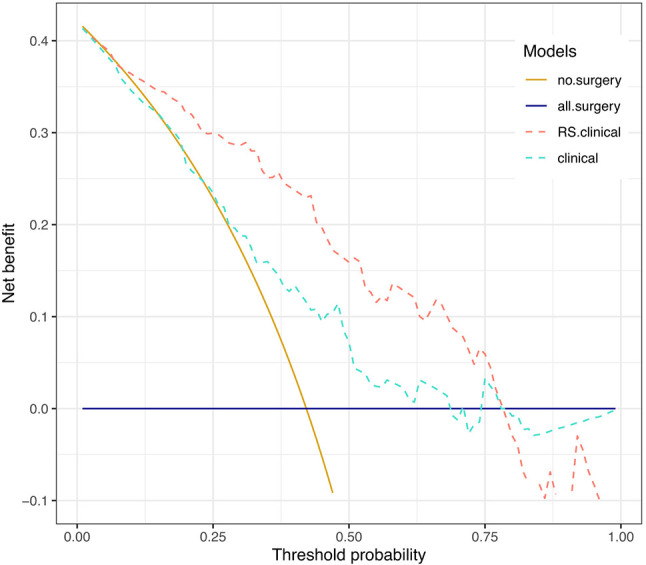
Decision curve analysis. Decision curves depicting the net benefit (y-axis) of the two models at a range of probability thresholds (i.e., minimum probability of pCR above which a patient would opt for observation rather than surgery; x-axis). The yellow and blue solid lines represent making the same decision in all patients (i.e., Sparing surgery for all patients or performing surgery for all patients, respectively). The net benefit was corrected by internal validation of 2,000-replicate bootstrap.

Net benefits of the two models were presented in **Figure 4**. Net benefit in our case is interpreted as the benefit of saving esophagus for pCR patients (true positive) who are correctly identified by the prediction model to spare surgery subtracting the harm of tumor residual in non-pCR patients (false positive) who are falsely judged by the model to omit operation. The horizontal solid line represents the clinical decision of preforming esophagectomy on all patients regardless of their response to NCRT, and it serves as a reference to visualize the benefit of treatment decisions by different models. When applying the RS+clinical model, a net benefit higher than that of the clinical model could be achieved at a threshold probability above 25%.

For example, at the 60% threshold cutoff (i.e., the patient would opt for observation if his probability of pCR was >60%), the net benefit was 0% in the all-surgery scheme, 2.23% in the clinical model, and 13% in the RS+clinical model, respectively. In other words, if we make treatment decision based on the RS+clinical model, the net benefit of 13% was equivalent to avoiding surgeries (taking organ-saving strategy) in 13 per 100 patients without an increase in the number of false-pCR predictions, which is a considerable gain compared with assuming that all patients have residual cancer and performing surgery for all patients. Overall, a total of 37 out of 121 patients (30.58%) could have been spared surgeries by RS+clinical model, while only 7 out of 70 patients (10%) with non-pCR would have been misdiagnosed.

**Figure 5:**
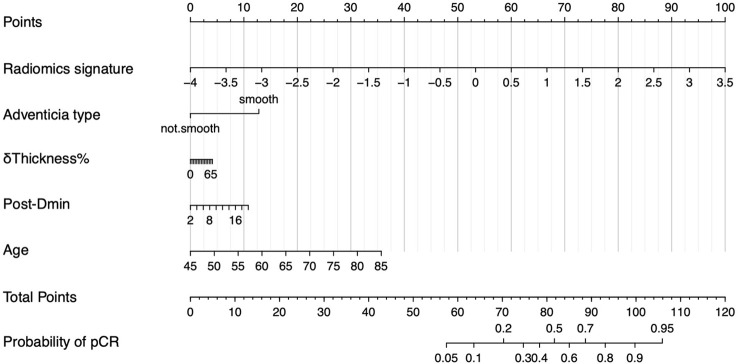
Nomogram of RS+clinical model. The nomogram built based on radiomics signature and clinical variables provide an easy-to-use tool in clinical practice.

To provide the clinician with a quantitative tool to predict individual probability of pCR, we built a nomogram based on the RS+clinical model (**Figure 5**).

## Discussion

We developed a prediction model for pCR to NCRT in ESCC using a CT-based radiomics signature and clinical variables. The model was internally validated and presented as a nomogram, showing satisfying performance in guiding clinical decision making.

Establishing a non-surgical approach to evaluate the tumor response to NCRT is crucial for making individualized treatment plans for locally advanced esophageal cancer. Esophagectomy is an effective intervention but comes with a high postoperative complication rate of roughly 65%, high postoperative mortality rate of 4%–10%, and decreased health-related quality of life, especially physical function that would never restore to pre-esophagectomy levels (6, 9, 10, 25). Patients who have an adequate response to NCRT, especially ESCC patients, of whom up to 43.2% could achieve pCR, might have a chance to spare surgery and preserve the esophagus (4).

In recent years, non-invasive radiomics analysis has been proven effective in prediction of tumor treatment response and patient survival. The underlying rationale is that tumor genetic heterogeneity will be converted to histopathological characteristics that can be reflected in medical images (13). Efforts have been made to predict tumor response to NCRT in esophageal cancer. Beukinga et al. (14) built a prediction model based on clinical T stage and joint maximum (a PET/CT radiomics parameter quantifying image orderliness) and achieved a corrected AUC of 0.81. van Rossum et al. (3) built a model consisting of total lesion glycolysis and four comprehensive ^18^F-FDG PET texture features with a corrected c-index of 0.77 but failed to find an incremental value in decision curve analysis. However, these studies focused primarily on esophageal adenocarcinoma, of which the tumor biologic characteristics as well as the response to NCRT are quite different from ESCC (pCR rate, 27% vs. 43.2%) (3, 4). The existing CT-based radiomics study aiming to predict NCRT response for ESCC contained only a small sample size ranging from 49 to 94 and were mostly unbalanced inregards to the pCR to non-pCR ratio, moreover, the previous studies produced relatively low model effectiveness (AUC of 0.54 ~ 0.79) (26–28). The research by Hu et al. (29) proves the feasibility of using CT radiomics to predict the treatment response of esophageal squamous cell cancer after chemoradiotherapy, but they fail to include traditional clinical and imaging data in the model. In the present study, a prediction model for pCR has been developed exclusively for ESCC, with a larger sample size (n=121) and a promising discriminative performance when uniting radiomics signature with clinical variables (AUC=0.843).

Comparing to PET-based radiomics model (3, 14, 30, 31), CT-based radiomics models have increasingly demonstrated non-inferior performance in NCRT response prediction, not only in ESCC as reported in the present study but also in other tumor types, such as rectal cancer (AUC=0.70) (32) and stage III non-small cell lung cancer (AUC=0.86) (33). Considering that CT is usually more accessible and affordable than PET for most cancer patients, it is reasonable to believe that a CT-based radiomics model is going to play an important role in NCRT response prediction and help to further personalize treatment strategies in multiple cancers. We also anticipate a robuster prediction potential if the model combines the CT and PET radiomics that we would further investigate in the future.

In our study, four clinical variables have exhibited significant association with pCR, including tumor adventitia type, δthickness% by CT, post-Dmin by esophagogram, and age. The value of tumor thickness derived parameters (percentage decrease, pre- or post-NCRT maximum tumor thickness, etc.) and the tumor outer membrane type in prediction of response to preoperative treatments has been investigated in previous studies (15, 34), but inconsistent conclusions were drawn. According to the study by Chee et al., the minimum luminal width on esophagogram has only moderate effectiveness in evaluating the tumor neoadjuvant treatment response when applied as a single predictive parameter (35). The limited usefulness of tumor thickness on CT and luminal width on esophagogram could be possibly explained by the bulking effect of necrotic and fibrotic tissues after neoadjuvant treatment, which results in the persistent abnormality on imaging tests. Radiomics is complementary to the traditional imaging parameters with its advantage to detect the heterogeneity within tissues, which makes it possible to improve the model performance in tumor response prediction. Interestingly, age was turned out to be related to the pCR status in our study with an OR of 1.08 (1.02, 1.16), indicating 1.08 times increase in the odds of pCR with per year increment in age. A similar finding was reported by Vandendorpe et al. (32) stating that age achieved an OR of 1.05 (1.00–1.10) in a model to evaluate the clinical downstaging of post-NCRT colorectal cancer. The potential biological or socio-economical causes behind this finding need to be further investigated.

The RS+clinical model exhibits the potential to categorize patients with different response to NCRT, according to which the treatment plan could be tailored to the individual situation. Patients who were predicted to have residual cancer will continue to receive esophagectomy. For those who are “radiomicly-determined” as potential pCR, surgery could be withheld and the organ-saving strategy could be taken, such as boosting the dose of radiotherapy to the definitive level or close surveillance (salvage surgery if necessary) after chemoradiation. Decision curve analysis proves that at a given threshold probability, using RS+clinical model to evaluate treatment response provides more clinical benefit than both clinical model-based strategy and all-surgery scheme. At the 60% threshold cutoff, net 13% surgeries could be avoided without an increase in the number of missed residual cancer by RS+clinical model. In other words, the correct pCR prediction of RS+clinical model would lead to a net reduction of 16 avoidable surgeries in the 121 patients of our research cohort, equivalent to performing organ-saving strategy in 31.37% of the 51 true-pCR cases. The threshold probability is not necessarily fixed at 0.6 in clinical practice and can be adjusted according to the patient’s individualized willingness to omit surgery. When it’s set to a stricter number higher than 0.6, the misdiagnosis rate will accordingly decline so the patient can take on less risk of tumor residue, though fewer patients can benefit from organ-saving treatment at the same time. Therefore, a balance needs to be struck between gaining net benefit and reducing misdiagnosis rate when determining the threshold probability.

When implementing organ-saving strategies, boosting the radiation dose might be a solution to reduce the potential risk of cancer recurrence in false-pCR patients, as supported by the results of several studies indicating that definitive chemoradiotherapy and trimodality treatment (NCRT followed by surgery) lead to similar survival outcome but the former accompanies with significantly lower treatment-related mortality rate (0.8%–3.5% vs. 9.3%–12.8%) (7, 36, 37). Close surveillance with necessary salvage esophagectomy has also been indicated feasible by previous studies. For example, Markar et al. (38) retrospectively analyzed 848 patients undergoing planned surgery after NCRT or salvage surgery after definitive chemoradiotherapy and found no significant difference in long-term survival as well as comparable short-term outcomes in selected patients at experienced centers. The ongoing prospective SANO trial and ESOSTRATE trial are investigating if active surveillance and surgery as needed after NCRT leads to non-inferior survival than standard esophagectomy (8, 39). If so, patients with an adequate response to NCRT identified by prediction models like the one presented in our study will be able to receive organ-saving treatments as a standard of care.

Several limitations apply to our study. First of all, this was a retrospective study with a relatively small study cohort, where division of training and testing set might cause bias, so the performance was corrected by internal validation of bootstrap. However, our study can be regarded as an exploratory effort that offers a theory foundation for future external validation on a larger scale. Second, previous studies included histopathologic grading of endoscopic biopsy in clinical variable analysis (3), but pre-NCRT biopsy specimens were only available in less than 1/3 patients of our cohort (most of which was taken outside our institution), so histopathologic grading was not included in our study. Third, PET parameters were not included in this retrospective study because only a small proportion of the patients received pre-NCRT or post-NCRT PETCT scan; however, we believe the additive value of PET will lead to the better performance of the predictive model, which we will explore in the future.

## Conclusion

We proposed a handy CT radiomics based model with satisfying performance to discriminate post-NCRT pCR patients from non-pCR ones. Clinical benefits introduced by the model may potentially facilitate individualized organ-preservation strategies on ESCC patients who have an adequate response to NCRT.

## Data Availability Statement

The raw data supporting the conclusions of this article will be made available by the authors, without undue reservation.

## Ethics Statement

The studies involving human participants were reviewed and approved by Shanghai Chest Hospital Institutional Review Board. Written informed consent for participation was not required for this study in accordance with the national legislation and the institutional requirements.

##  Author Contributions

YL: Conceptualization, data curation, formal analysis, investigation, methodology, software, validation, visualization, writing—original draft. JL: Data curation, methodology, resources, writing—review and editing. X-WC: Investigation, methodology, resources. H-XL: Methodology, resources. Z-GL: Resources. X-DY: Methodology. H-HT: Methodology. WY: Conceptualization, funding acquisition, methodology, project administration, supervision, writing—review and editing. X-LF: Conceptualization, methodology, project administration, resources, supervision, writing—review and editing. All authors contributed to the article and approved the submitted version.

## Funding

Shanghai Pujiang Program (18PJD046); Shanghai Chest Hospital Project of Collaborative Innovation (YJXT20190202Z).

## Conflict of Interest

The authors declare that the research was conducted in the absence of any commercial or financial relationships that could be construed as a potential conflict of interest.
