# Supplementary material for: Radiomics Signature Facilitates Organ-Saving Strategy in Patients With Esophageal Squamous Cell Cancer Receiving Neoadjuvant Chemoradiotherapy
Source: Front Oncol. 2021 Feb 19;10:615167. doi: 10.3389/fonc.2020.615167 (PMC7933499; doi:10.3389/fonc.2020.615167)
Supplement: Supplementary file 1 [file DataSheet_1.docx]

**Supplementary Materials**

**1.The general principle of radiomics feature extraction**

**2.Features with good reproducibility in inter-observer test(intraclass correlation coefficient, ICC>0.4) and test-retest(concordance correlation coefficient, CCC>0.75).**

**3. Radiomics feature preselection - univariate logistic regression analysis results.**

**4.The 18 selected radiomics features and formula for RS calculation.**

**5. Calculation formula for selected features**

**6.feature correlation matrix & boxplot**

**7.The formula of pCR probability calculation from the clinical model and the RS+clinical model.**

**1.The general principle of radiomics feature extraction**

We extracted 135 features from pre-NCRT and post-NCRT CT images, respectively, which are divided into 3 groups:

1) Shape based features

This group includes 18 features that describe the volume, surface area, compactness and other three dimentional shape and size derived characteristics of the delineated tumor.

2)First order statistics

This group contains descriptors of pixel/voxel grey level distribution throughout the image ROI (region of interest), but they don’t take into acount the pixel/voxel location or their spatial relationship. 52 features belong to this group, including parameters calculated based on intensity histogram (kurtosis, skewness, percentile area value, etc.) and direct intensity features (global mean, global entropy, etc.).

3)Second order statistics

This group consists 65 textural features that take pixel/voxel spatial relationship into consideration. Three subgroups are included: neighbor intensity difference(NID) depicts image texture including busyness, coarseness, complexity, contrast, and texture strength based on neighboring pixel grey tone intensity;^1^ gray level cooccurrence matrix(GLCM) describes the joint probability of two pixels with a specific pair of gray level occur in an image; gray level run length matrix(GLRLM) is composed of the count of consecutive pixels/voxels with identical gray level at different directions. ^2,3^ NID and GLCM are calculated in both two and a half dimension (2.5D) and 3D versions.

Images were pre-processed by image masking with a lower boundary of 50 Hounsfield(HU) to exclude the esophageal luminal air and an upper boundary of 1400 HU to eliminate image blocks of calcification and titanium clips placed to facilitate the intraoperative localization of the tumors. For both the first and the second order statistic features, an 8-bit depth resampling was used, setting a bin width of 16 HU, to reduce noise in the image and normalize intensities across all patients.^4^

**2.Features with good reproducibility in inter-observer test(intraclass correlation coefficient, ICC>0.4) and test-retest(concordance correlation coefficient, CCC>0.75).**

| radiomics | ICC | lbound | ubound |  | CCC | lbound | ubound |
| --- | --- | --- | --- | --- | --- | --- | --- |
| VoxelSize-F1 | 1.000 | NaN | NaN |  | 0.920 | 0.834 | 0.962 |
| LocalRangeMin-F5 | 1.000 | NaN | NaN |  | 0.813 | 0.685 | 0.893 |
| LocalStdMin-F5 | 1.000 | NaN | NaN |  | 0.804 | 0.650 | 0.894 |
| SurfaceAreaDensity-F1 | 0.917 | 0.717 | 0.978 |  | 0.986 | 0.971 | 0.993 |
| LocalStdMean-F5 | 0.914 | 0.705 | 0.978 |  | 0.972 | 0.941 | 0.987 |
| 75Percentile-F4 | 0.880 | 0.584 | 0.969 |  | 0.950 | 0.894 | 0.977 |
| 0.75Quantile-F4 | 0.880 | 0.584 | 0.969 |  | 0.950 | 0.894 | 0.977 |
| 75Percentile-F5 | 0.880 | 0.584 | 0.969 |  | 0.950 | 0.894 | 0.977 |
| 0.75Quantile-F5 | 0.880 | 0.584 | 0.969 |  | 0.950 | 0.894 | 0.977 |
| LocalRangeMean-F5 | 0.879 | 0.464 | 0.971 |  | 0.969 | 0.934 | 0.985 |
| -333-1InverseVariance-F8 | 0.860 | 0.555 | 0.963 |  | 0.932 | 0.858 | 0.969 |
| -333LongRunLowGrayLevelEmpha-F6 | 0.852 | 0.511 | 0.961 |  | 0.912 | 0.818 | 0.959 |
| -333LongRunEmphasis-F6 | 0.849 | 0.501 | 0.960 |  | 0.916 | 0.824 | 0.960 |
| -333LongRunHighGrayLevelEmpha-F6 | 0.849 | 0.499 | 0.960 |  | 0.917 | 0.827 | 0.961 |
| LocalRangeStd-F5 | 0.846 | 0.518 | 0.959 |  | 0.963 | 0.926 | 0.982 |
| -333-1InformationMeasureCorr1-F8 | 0.836 | 0.371 | 0.959 |  | 0.791 | 0.598 | 0.897 |
| Compactness1-F1 | 0.794 | -0.063 | 0.959 |  | 0.992 | 0.982 | 0.996 |
| LocalEntropyMedian-F5 | 0.767 | -0.042 | 0.949 |  | 0.968 | 0.932 | 0.985 |
| LocalStdStd-F5 | 0.761 | 0.208 | 0.938 |  | 0.927 | 0.849 | 0.965 |
| LocalRangeMedian-F5 | 0.737 | 0.124 | 0.932 |  | 0.979 | 0.954 | 0.990 |
| Coarseness-F3 | 0.730 | -0.070 | 0.940 |  | 0.935 | 0.864 | 0.969 |
| LocalStdMedian-F5 | 0.730 | 0.132 | 0.929 |  | 0.980 | 0.956 | 0.991 |
| Orientation-F1 | 0.718 | 0.064 | 0.928 |  | 0.753 | 0.514 | 0.873 |
| RootMeanSquare-F5 | 0.715 | 0.047 | 0.927 |  | 0.945 | 0.883 | 0.974 |
| Roundness-F1 | 0.712 | 0.170 | 0.921 |  | 0.766 | 0.563 | 0.883 |
| 50Percentile-F4 | 0.706 | 0.048 | 0.924 |  | 0.949 | 0.893 | 0.977 |
| 0.5Quantile-F4 | 0.706 | 0.048 | 0.924 |  | 0.949 | 0.893 | 0.977 |
| GlobalMedian-F5 | 0.706 | 0.048 | 0.924 |  | 0.949 | 0.893 | 0.977 |
| 50Percentile-F5 | 0.706 | 0.048 | 0.924 |  | 0.949 | 0.893 | 0.977 |
| 0.5Quantile-F5 | 0.706 | 0.048 | 0.924 |  | 0.949 | 0.893 | 0.977 |
| -333-1InverseDiffNorm-F8 | 0.696 | 0.199 | 0.913 |  | 0.927 | 0.852 | 0.965 |
| 50PercentileArea-F4 | 0.696 | 0.204 | 0.913 |  | 0.955 | 0.905 | 0.979 |
| -333-1AutoCorrelation-F7 | 0.677 | 0.130 | 0.909 |  | 0.929 | 0.852 | 0.967 |
| GlobalMean-F5 | 0.672 | -0.006 | 0.914 |  | 0.940 | 0.874 | 0.972 |
| -333HighGrayLevelRunEmpha-F6 | 0.670 | 0.137 | 0.905 |  | 0.844 | 0.690 | 0.925 |
| -333-1SumAverage-F7 | 0.663 | 0.110 | 0.904 |  | 0.929 | 0.852 | 0.967 |
| Contrast-F3 | 0.648 | 0.091 | 0.899 |  | 0.893 | 0.795 | 0.946 |
| LocalEntropyMean-F5 | 0.645 | -0.095 | 0.914 |  | 0.968 | 0.931 | 0.985 |
| Energy-F5 | 0.636 | -0.094 | 0.909 |  | 0.986 | 0.974 | 0.992 |
| -333RunPercentage-F6 | 0.635 | -0.076 | 0.906 |  | 0.953 | 0.902 | 0.978 |
| NumberOfVoxel-F1 | 0.630 | -0.097 | 0.908 |  | 0.986 | 0.974 | 0.993 |
| Volume-F1 | 0.611 | -0.102 | 0.902 |  | 0.989 | 0.977 | 0.995 |
| Mass-F1 | 0.610 | -0.102 | 0.901 |  | 0.990 | 0.979 | 0.995 |
| 25PercentileArea-F4 | 0.610 | -0.049 | 0.892 |  | 0.934 | 0.862 | 0.970 |
| 75PercentileArea-F4 | 0.600 | 0.059 | 0.879 |  | 0.955 | 0.905 | 0.979 |
| TextureStrength-F3 | 0.593 | -0.017 | 0.882 |  | 0.875 | 0.745 | 0.941 |
| 25Percentile-F4 | 0.580 | -0.067 | 0.881 |  | 0.929 | 0.852 | 0.967 |
| 0.25Quantile-F4 | 0.580 | -0.067 | 0.881 |  | 0.929 | 0.852 | 0.967 |
| 25Percentile-F5 | 0.580 | -0.067 | 0.881 |  | 0.929 | 0.852 | 0.967 |
| 0.25Quantile-F5 | 0.580 | -0.067 | 0.881 |  | 0.929 | 0.852 | 0.967 |
| ConvexHullVolume-F1 | 0.565 | -0.110 | 0.885 |  | 0.985 | 0.968 | 0.993 |
| -333-1InverseDiffMomentNorm-F8 | 0.561 | -0.030 | 0.867 |  | 0.884 | 0.777 | 0.942 |
| -333-1MaxProbability-F7 | 0.558 | -0.094 | 0.875 |  | 0.956 | 0.907 | 0.980 |
| -333-1Energy-F7 | 0.550 | -0.098 | 0.872 |  | 0.953 | 0.901 | 0.978 |
| -333-1InverseVariance-F7 | 0.546 | -0.108 | 0.873 |  | 0.941 | 0.875 | 0.972 |
| -333-1ClusterTendendcy-F7 | 0.544 | -0.035 | 0.860 |  | 0.839 | 0.678 | 0.923 |
| -333-1Variance-F7 | 0.544 | -0.035 | 0.860 |  | 0.839 | 0.678 | 0.923 |
| -333-1Homogeneity-F7 | 0.538 | -0.100 | 0.867 |  | 0.940 | 0.875 | 0.972 |
| -333-1Homogeneity2-F7 | 0.537 | -0.097 | 0.865 |  | 0.939 | 0.874 | 0.971 |
| -333-1Dissimilarity-F7 | 0.533 | -0.094 | 0.863 |  | 0.932 | 0.861 | 0.967 |
| -333-1Homogeneity-F8 | 0.532 | -0.094 | 0.878 |  | 0.931 | 0.857 | 0.968 |
| -333-1InverseDiffNorm-F7 | 0.531 | -0.096 | 0.862 |  | 0.932 | 0.861 | 0.967 |
| -333-1Homogeneity2-F8 | 0.528 | -0.093 | 0.876 |  | 0.931 | 0.856 | 0.967 |
| -333-1SumVariance-F7 | 0.520 | -0.107 | 0.859 |  | 0.953 | 0.899 | 0.978 |
| -333-1Contrast-F7 | 0.515 | -0.082 | 0.851 |  | 0.901 | 0.813 | 0.948 |
| InterQuartileRange-F4 | 0.509 | -0.096 | 0.851 |  | 0.920 | 0.835 | 0.962 |
| InterQuartileRange-F5 | 0.509 | -0.096 | 0.851 |  | 0.920 | 0.835 | 0.962 |
| -333-1DifferenceEntropy-F7 | 0.508 | -0.113 | 0.855 |  | 0.932 | 0.857 | 0.968 |
| -333-1Entropy-F7 | 0.502 | -0.114 | 0.852 |  | 0.951 | 0.896 | 0.977 |
| LocalEntropyStd-F5 | 0.499 | -0.112 | 0.859 |  | 0.956 | 0.907 | 0.980 |
| -333-1SumEntropy-F7 | 0.498 | -0.116 | 0.851 |  | 0.946 | 0.886 | 0.974 |
| -333GrayLevelNonuniformity-F6 | 0.495 | -0.118 | 0.851 |  | 0.981 | 0.963 | 0.990 |
| Kurtosis-F4 | 0.486 | -0.167 | 0.842 |  | 0.978 | 0.953 | 0.990 |
| Kurtosis-F5 | 0.486 | -0.167 | 0.842 |  | 0.978 | 0.953 | 0.990 |
| -333-1DifferenceEntropy-F8 | 0.481 | -0.103 | 0.853 |  | 0.935 | 0.863 | 0.969 |
| SurfaceArea-F1 | 0.480 | -0.115 | 0.849 |  | 0.979 | 0.955 | 0.991 |
| -333-1Dissimilarity-F8 | 0.467 | -0.115 | 0.843 |  | 0.927 | 0.852 | 0.965 |
| Max3DDiameter-F1 | 0.463 | -0.122 | 0.835 |  | 0.972 | 0.943 | 0.987 |
| -333-1ClusterShade-F7 | 0.460 | -0.088 | 0.822 |  | 0.864 | 0.734 | 0.932 |
| ConvexHullVolume3D-F1 | 0.458 | -0.091 | 0.821 |  | 0.981 | 0.959 | 0.991 |
| -333-1InverseDiffMomentNorm-F7 | 0.438 | -0.140 | 0.815 |  | 0.871 | 0.758 | 0.933 |
| MeanBreadth-F1 | 0.433 | -0.124 | 0.820 |  | 0.969 | 0.933 | 0.985 |
| -333-1ClusterTendendcy-F8 | 0.433 | -0.116 | 0.811 |  | 0.829 | 0.662 | 0.918 |
| -333-1Variance-F8 | 0.433 | -0.116 | 0.811 |  | 0.829 | 0.662 | 0.918 |
| -333-1MaxProbability-F8 | 0.429 | -0.081 | 0.831 |  | 0.953 | 0.900 | 0.978 |
| MeanAbsoluteDeviation-F4 | 0.415 | -0.117 | 0.814 |  | 0.932 | 0.862 | 0.967 |
| MeanAbsoluteDeviation-F5 | 0.415 | -0.117 | 0.814 |  | 0.932 | 0.862 | 0.967 |
| Coarseness-F2 | 0.411 | -0.121 | 0.810 |  | 0.835 | 0.791 | 0.870 |
| Variance-F5 | 0.407 | -0.127 | 0.800 |  | 0.889 | 0.783 | 0.945 |

Feature index number reference: F1-Shape; F2-NeighborIntensityDifference3; F3-NeighborIntensityDifference25; F4-IntensityHistogram; F5-IntensityDirect; F6-GrayLevelRunLengthMatrix25; F7-GrayLevelCooccurenceMatrix3; F8-GrayLevelCooccurenceMatrix25

**3. Radiomics feature preselection - univariate logistic regression analysis results.**

| Radiomics feature name | Coefficient estimate | Std. Error | z value | Pr(>\|z\|) |
| --- | --- | --- | --- | --- |
| preLocalRangeMin-F5 | 2.144 | 0.760 | 2.822 | 0.005 |
| preLocalStdMin-F5 | 1.992 | 0.768 | 2.593 | 0.010 |
| pre-333-1InverseVariance-F8 | 2.168 | 1.164 | 1.863 | 0.062 |
| pre-333-1InformationMeasureCorr1-F8 | 2.954 | 1.178 | 2.507 | 0.012 |
| preCoarseness-F3 | -1.997 | 0.892 | -2.240 | 0.025 |
| preOrientation-F1 | 1.970 | 1.103 | 1.787 | 0.074 |
| pre-333-1InverseDiffNorm-F8 | -2.159 | 1.109 | -1.946 | 0.052 |
| pre-333-1InverseDiffMomentNorm-F8 | -2.013 | 0.993 | -2.027 | 0.043 |
| pre-333-1Homogeneity-F8 | -2.167 | 1.089 | -1.989 | 0.047 |
| pre-333-1Homogeneity2-F8 | -2.092 | 1.077 | -1.942 | 0.052 |
| pre-333-1Dissimilarity-F8 | 1.559 | 1.031 | 1.512 | 0.130 |
| pre-333-1MaxProbability-F8 | -2.320 | 1.446 | -1.604 | 0.109 |
| postVoxelSize-F1 | -2.102 | 1.420 | -1.480 | 0.139 |
| postSurfaceAreaDensity-F1 | 3.091 | 1.402 | 2.205 | 0.027 |
| post75Percentile-F4 | -2.437 | 1.327 | -1.837 | 0.066 |
| post0.75Quantile-F4 | -2.437 | 1.327 | -1.837 | 0.066 |
| post75Percentile-F5 | -2.437 | 1.327 | -1.837 | 0.066 |
| post0.75Quantile-F5 | -2.437 | 1.327 | -1.837 | 0.066 |
| postCompactness1-F1 | -2.729 | 1.597 | -1.709 | 0.087 |
| postOrientation-F1 | 2.238 | 1.293 | 1.731 | 0.083 |
| postRootMeanSquare-F5 | -2.468 | 1.314 | -1.878 | 0.060 |
| post50Percentile-F4 | -3.271 | 1.555 | -2.103 | 0.035 |
| post0.5Quantile-F4 | -3.271 | 1.555 | -2.103 | 0.035 |
| postGlobalMedian-F5 | -3.271 | 1.555 | -2.103 | 0.035 |
| post50Percentile-F5 | -3.271 | 1.555 | -2.103 | 0.035 |
| post0.5Quantile-F5 | -3.271 | 1.555 | -2.103 | 0.035 |
| postGlobalMean-F5 | -2.365 | 1.445 | -1.637 | 0.102 |
| postMass-F1 | -2.291 | 1.593 | -1.438 | 0.151 |
| post75PercentileArea-F4 | -2.915 | 1.658 | -1.758 | 0.079 |
| post25Percentile-F4 | -2.365 | 1.245 | -1.900 | 0.057 |
| post0.25Quantile-F4 | -2.365 | 1.245 | -1.900 | 0.057 |
| post25Percentile-F5 | -2.365 | 1.245 | -1.900 | 0.057 |
| post0.25Quantile-F5 | -2.365 | 1.245 | -1.900 | 0.057 |
| deltLocalRangeMin-F5 | 1.664 | 0.592 | 2.811 | 0.005 |
| deltLocalStdMin-F5 | 1.497 | 0.604 | 2.479 | 0.013 |
| deltSurfaceAreaDensity-F1 | -2.040 | 1.168 | -1.747 | 0.081 |
| delt-333-1InformationMeasureCorr1-F8 | 1.610 | 0.685 | 2.351 | 0.019 |
| deltCompactness1-F1 | 2.544 | 1.489 | 1.709 | 0.087 |
| deltCoarseness-F3 | -1.910 | 0.770 | -2.479 | 0.013 |
| deltRootMeanSquare-F5 | 1.507 | 0.976 | 1.543 | 0.123 |
| delt50Percentile-F4 | 1.706 | 0.888 | 1.921 | 0.055 |
| delt0.5Quantile-F4 | 1.706 | 0.888 | 1.921 | 0.055 |
| deltGlobalMedian-F5 | 1.706 | 0.888 | 1.921 | 0.055 |
| delt50Percentile-F5 | 1.706 | 0.888 | 1.921 | 0.055 |
| delt0.5Quantile-F5 | 1.706 | 0.888 | 1.921 | 0.055 |
| delt25Percentile-F4 | 1.364 | 0.909 | 1.500 | 0.134 |
| delt0.25Quantile-F4 | 1.364 | 0.909 | 1.500 | 0.134 |
| delt25Percentile-F5 | 1.364 | 0.909 | 1.500 | 0.134 |
| delt0.25Quantile-F5 | 1.364 | 0.909 | 1.500 | 0.134 |

Feature index number reference: F1-Shape; F2-NeighborIntensityDifference3; F3-NeighborIntensityDifference25; F4-IntensityHistogram; F5-IntensityDirect; F6-GrayLevelRunLengthMatrix25; F7-GrayLevelCooccurenceMatrix3; F8-GrayLevelCooccurenceMatrix25

Prefix of “pre“, “delt“, “post“ refer to pre-NCRT, δ-NCRT, post-NCRT radiomics features, respectively.

**4.The 18 selected radiomics features and formula for RS calculation.**

**RS** = 0.27*preLocalRangeMin-F5

1.81*pre-333-1InverseVariance-F8

0.32*pre-333-1InformationMeasureCorr1-F8

0.77*preOrientation-F1

-0.66*pre-333-1InverseDiffMomentNorm-F8

-0.94*postVoxelSize-F1

2.06*postSurfaceAreaDensity-F1

-0.93*post75Percentile-F4

-0.2*post0.75Quantile-F4

1.7*postOrientation-F1

-0.12*post50Percentile-F4

-1.81*post75PercentileArea-F4

0.34*deltLocalRangeMin-F5

-1.25*deltSurfaceAreaDensity-F1

0.07*delt-333-1InformationMeasureCorr1-F8

0.51*deltCompactness1-F1

-1.41*deltCoarseness-F3

1.18*delt50Percentile-F4

Feature index number reference: F1-Shape; F2-NeighborIntensityDifference3; F3-NeighborIntensityDifference25; F4-IntensityHistogram; F5-IntensityDirect; F6-GrayLevelRunLengthMatrix25; F7-GrayLevelCooccurenceMatrix3; F8-GrayLevelCooccurenceMatrix25

Prefix of “pre“, “delt“, “post“ refer to pre-NCRT, δ-NCRT, post-NCRT radiomics features, respectively.

**5. Calculation formula for selected features**

**Group 1. Shape based radiomics features**

1) Compactness1:^2^

Let V denotes the volume, and A refers to the surface area:

$\boldsymbol{compactness}\boldsymbol{1}\mathbf{=}\frac{\boldsymbol{V}}{\sqrt{\boldsymbol{\pi}}\boldsymbol{A}^{\frac{\boldsymbol{2}}{\boldsymbol{3}}}}$

**Group 2. First order statistics**

2)Local range: Range refers tothe range of intensity values of the three dimensional image matrix.^2^

**Group 3. Second order statistics**

Let:

***P(i,j)*** be the co-occurrence matrix for an arbitrary and be the number of discrete ,

***N_g_*** be the intensity levels in the image,

$\boldsymbol{p}_{\boldsymbol{x}}=\sum_{\boldsymbol{j=1}}^{\boldsymbol{N}_{\boldsymbol{g}}} \boldsymbol{P(i,j)}$ be the marginal row probabilities,

$\boldsymbol{p}_{\boldsymbol{y}}=\sum_{\boldsymbol{i=1}}^{\boldsymbol{N}_{\boldsymbol{g}}} \boldsymbol{P(i,j)}$ be the marginal column probabilities.

3)InverseVariance:^2^

$$\boldsymbol{inverse variance=}\sum_{\boldsymbol{i=0}}^{\boldsymbol{N}_{\boldsymbol{g}}} \sum_{\boldsymbol{j=0}}^{\boldsymbol{N}_{\boldsymbol{g}}} \frac{\boldsymbol{P(i,j)}}{\left| \boldsymbol{i-j} \right|^{\boldsymbol{2}}}\boldsymbol{,i\neq j}$$

4) Informational measure of correlation 2 (IMC2):^2^

$$IMC2=\sqrt{1-e^{-2(HXY2-HXY)}}$$

,where $HXY=\sum_{\boldsymbol{i=0}}^{\boldsymbol{N}_{\boldsymbol{g}}} \sum_{\boldsymbol{j=0}}^{\boldsymbol{N}_{\boldsymbol{g}}} \boldsymbol{P(i,j)log(}\boldsymbol{p}_{\boldsymbol{x}}\boldsymbol{(i)}\boldsymbol{p}_{\boldsymbol{y}}\boldsymbol{(j))}$

**,**and $HXY2=\sum_{\boldsymbol{i=0}}^{\boldsymbol{N}_{\boldsymbol{g}}} \sum_{\boldsymbol{j=0}}^{\boldsymbol{N}_{\boldsymbol{g}}} \boldsymbol{p}_{\boldsymbol{x}}\boldsymbol{(i)}\boldsymbol{p}_{\boldsymbol{y}}\boldsymbol{(j)log(}\boldsymbol{p}_{\boldsymbol{x}}\boldsymbol{(i)}\boldsymbol{p}_{\boldsymbol{y}}\boldsymbol{(j))}$**.**

5)Inverse Difference Moment Normalized (IDMN):^2^

$$\boldsymbol{inverse variance=}\sum_{\boldsymbol{i=0}}^{\boldsymbol{N}_{\boldsymbol{g}}} \sum_{\boldsymbol{j=0}}^{\boldsymbol{N}_{\boldsymbol{g}}} \frac{\boldsymbol{P(i,j)}}{\boldsymbol{1+(}\frac{\left| \boldsymbol{i-j} \right|^{\boldsymbol{2}}}{\boldsymbol{N}^{\boldsymbol{2}}}\boldsymbol{)}}$$

6)Texture strength: ^1^

$\boldsymbol{f}_{\boldsymbol{str}}\boldsymbol{=}\frac{\left[ \sum_{\boldsymbol{i=0}}^{\boldsymbol{N}_{\boldsymbol{g}}} \sum_{\boldsymbol{j=0}}^{\boldsymbol{N}_{\boldsymbol{g}}} \boldsymbol{(}\boldsymbol{p}_{\boldsymbol{i}}\boldsymbol{+}\boldsymbol{p}_{\boldsymbol{j}}\boldsymbol{)}\boldsymbol{(i-j)}^{\boldsymbol{2}} \right]}{\left[ \text{ε+}\sum_{\boldsymbol{i=0}}^{\boldsymbol{N}_{\boldsymbol{g}}} \boldsymbol{s(i)} \right]}$**,p_i_≠0, p_j_≠0.**

7)Coarseness: ^1^

$$\boldsymbol{f}_{\boldsymbol{cos}}\boldsymbol{=}\left[ \text{ε+}\sum_{\boldsymbol{i=0}}^{\boldsymbol{N}_{\boldsymbol{g}}} \boldsymbol{p}_{\boldsymbol{i}}\boldsymbol{s(i)} \right]^{\boldsymbol{-1}}$$

**6.feature correlation matrix & boxplot**

**Box plot of RS in pCR and non -pCR groups.**


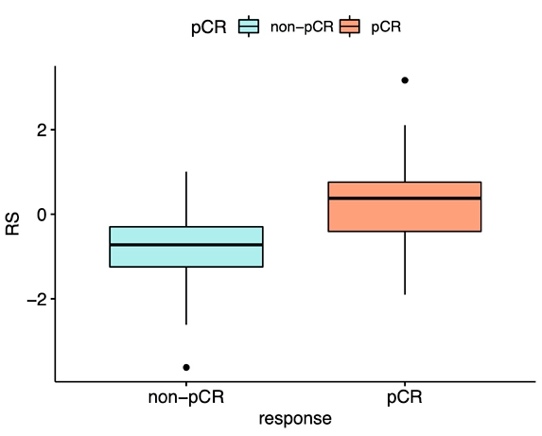


Boxplot of RS in pCR and non-pCR groups indicating an appearant mean difference.

**7.The formula of pCR probability calculation from the clinical model and the RS+clinical model.**

**1)RS+clinical model:**

$p=\frac{e^{1.62*RS+1.56*adventicia1+0.71*delthick.perc+0.07*Desogram.post+0.11*age-8.47}}{1+e^{1.62*RS+1.56*adventicia1+0.71*delthick.perc+0.07*Desogram.post+0.11*age-8.47}}$

**2)Clinical model**

**1.02*Adventitia type+2.32*δThickness% +0.11*Post-Dmin +0.07*Age -7.8**

$$p=\frac{e^{1.02*Adventitia type+2.32*\delta Thickness\% +0.11*Post-Dmin +0.07*Age -7.8}}{1+e^{1.02*Adventitia type+2.32*\delta Thickness\% +0.11*Post-Dmin +0.07*Age -7.8}}$$

“p” refers to the the probability of pCR.

*Reference:*

*1. Amadasun M, King R. Textural features corresponding to textural properties. IEEE Transactions on Systems, Man, and Cybernetics. 1989;19(5):1264-1274. doi:10.1109/21.44046*

*2. Aerts HJWL, Velazquez ER, Leijenaar RTH, et al. Decoding tumour phenotype by noninvasive imaging using a quantitative radiomics approach. Nature Communications. 2014;5(1). doi:10.1038/ncomms5006*

*3. Lubner MG, Smith AD, Sandrasegaran K, Sahani DV, Pickhardt PJ. CT Texture Analysis: Definitions, Applications, Biologic Correlates, and Challenges. RadioGraphics. 2017;37(5):1483-1503. doi:10.1148/rg.2017170056*

*4. Fave X, Zhang L, Yang J, et al. Impact of image preprocessing on the volume dependence and prognostic potential of radiomics features in non-small cell lung cancer. Translational Cancer Research. 2016;5(4):349-363. doi:10.21037/tcr.2016.07.11*
