# Supplementary figures and images for: Radiomics Signature Facilitates Organ-Saving Strategy in Patients With Esophageal Squamous Cell Cancer Receiving Neoadjuvant Chemoradiotherapy
Source: Front Oncol. 2021 Feb 19;10:615167. doi: 10.3389/fonc.2020.615167 (PMC7933499; doi:10.3389/fonc.2020.615167)

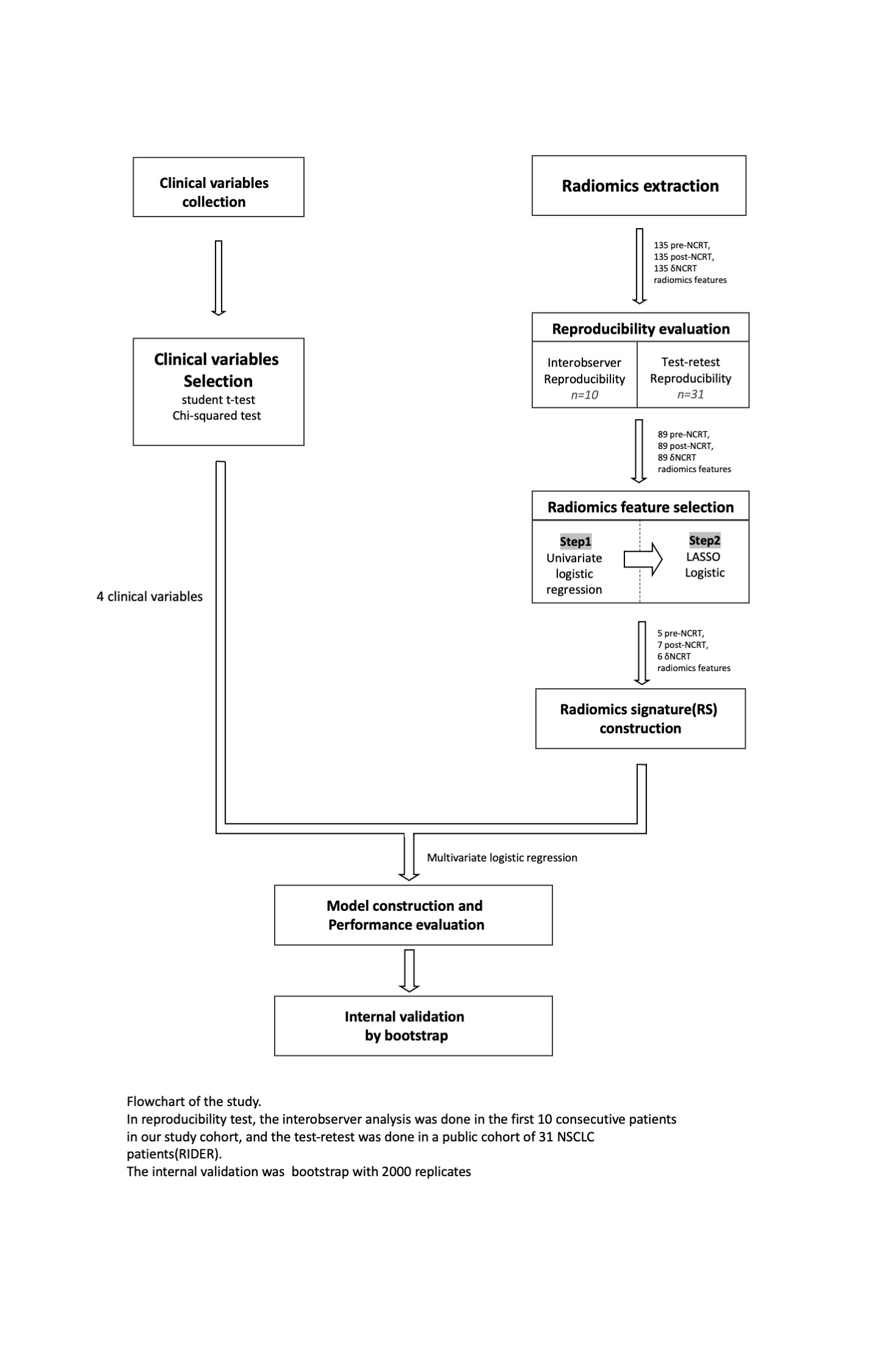

Supplement: Supplementary file 2 [file Image_1.jpeg]
